# Mandibular Atrophy and Genial Spines Enlargement on Cone Beam Computed Tomography

**DOI:** 10.1155/2014/803572

**Published:** 2014-07-23

**Authors:** Marcelo Lupion Poleti, Christiano Oliveira-Santos, Luciana Maria Paes da Silva Ramos Fernandes, Izabel Regina Fischer Rubira-Bullen

**Affiliations:** ^1^University of North Paraná (UNOPAR), Rua Marselha 183, 86041-140 Londrina, PR, Brazil; ^2^Paraná Federal Institute of Education, Science and Technology, Rua João XXIII 600, Jardim Dom Bosco, 86060-370 Londrina, PR, Brazil; ^3^Department of Morphology, Stomatology and Physiology, Ribeirão Preto School of Dentistry, University of Sao Paulo, Avenida do Café, s/n, Bairro Monte Alegre, 14040-904 Ribeirão Preto, SP, Brazil; ^4^Department of Stomatology, Bauru School of Dentistry, University of Sao Paulo, Alameda Otávio Pinheiro Brisolla, 9-75 Vila Universitária, 17012-901 Bauru, SP, Brazil

## Abstract

*Purpose*. The aim of this paper is to report a case in which the cone beam computed tomography (CBCT) was important for the confirmation of the presence of mandibular atrophy and genial spines enlargement. *Case Description*. A 76-year-old female patient was referred for the assessment due to the complaint of chronic trauma in the anterior region of the floor of the mouth, which had been present for 2 months. CBCT images showed severe resorption of alveolar ridge and genial spines enlargement (5.5 mm × 12 mm). *Conclusion*. Accurate imaging assessment with the aid of 3D reconstructions allows the elimination of image superimposition and, therefore, plays an important role in the depiction of anatomical and pathological conditions, such as genial spines enlargement.

## 1. Introduction

Cone beam computed tomography (CBCT) is a relatively new technology used for the three-dimensional (3D) imaging of the hard tissues of the maxillofacial region [1, 2]. This technology, when compared to conventional computed tomography (CT), has many advantages, such as radiation dose reduction, higher spatial resolution, and rapid scan time [1]. CT in general provides 3D imaging and has been used to overcome the inherent problems with conventional two-dimensional radiographic techniques [2], for example, superimposition of structures. Moreover, it is possible to obtain 3D reconstructions, which may improve the visualization of the assessed region.

Genial spines consist of a group of four bony extensions situated in the lingual aspect of the anterior region of the mandible, surrounding the lingual foramen [3]. The superior and inferior genial spines provide attachment for the genioglossus muscle and the geniohyoid muscle, respectively [4].

This paper reports a case in which CBCT 3D imaging was used to show mandibular atrophy and abnormal size of the genial spines in a patient wearing a complete denture.

## 2. Case Presentation

**Figure 1:**
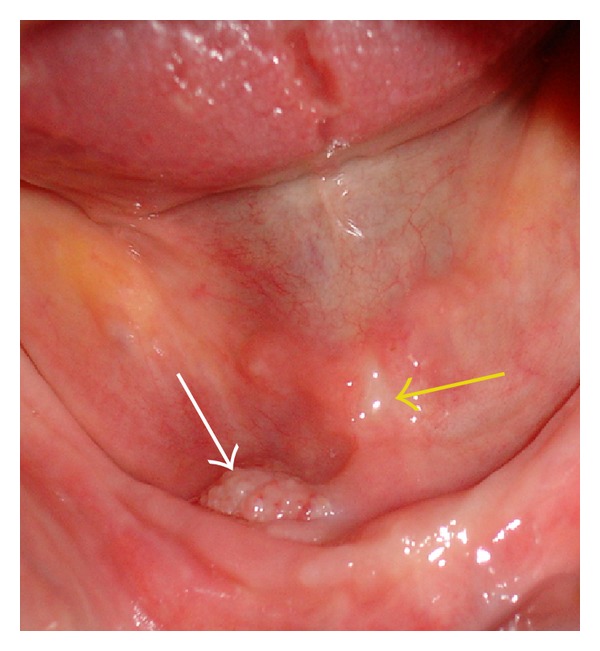
Intraoral view showing inflammatory fibrous hyperplasia (white arrow) and area of fibrosis of the floor of the mouth mucosa (yellow arrow).

**Figure 2:**
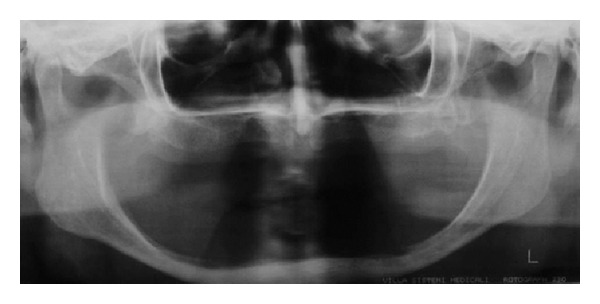
Panoramic radiograph showing severe resorption of mandibular alveolar ridge.

A 76-year-old Caucasian female was referred to our stomatology clinic due to the complaint of chronic trauma in the anterior region of the floor of the mouth, which had been present for 2 months. The patient reported the use of the same complete denture for five years, which was associated with pain when chewing. Local inspection revealed inflammatory fibrous hyperplasia and fibrosis of the floor of the mouth mucosa associated with unstable prosthesis (Figure 1). Panoramic radiograph revealed severe resorption of mandibular alveolar ridge (Figure 2).

**Figure 3:**
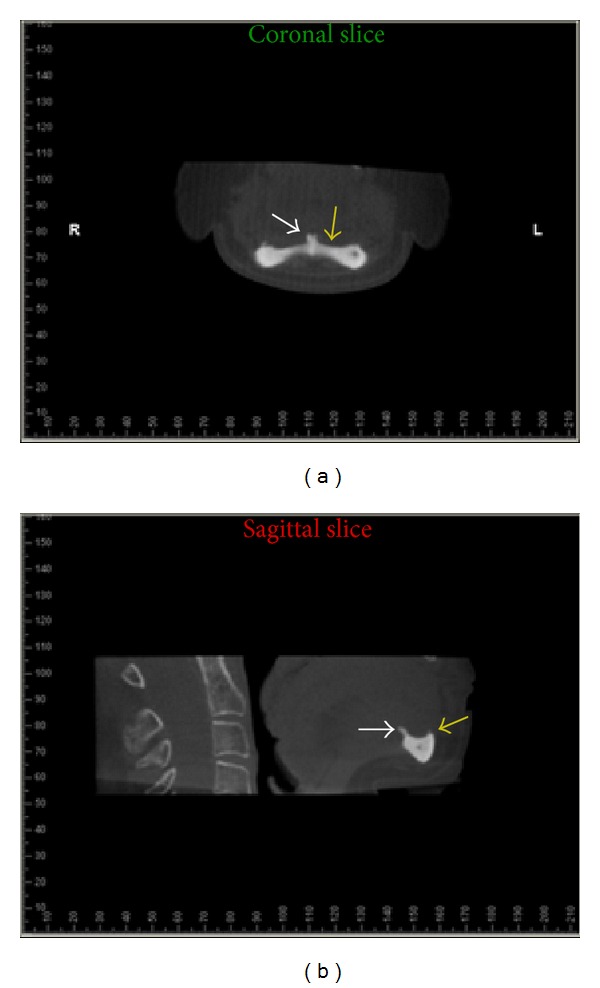
Coronal and sagittal slices showing severe resorption of alveolar ridge (yellow arrows) and increased genial spine (white arrows).

In order to better visualize the region a CBCT scan was performed (i-CAT Classic/Imaging Sciences International, Hatfield, PA, USA), using 6 cm FOV, 20 seconds, and 0.3 mm voxel size. The images were reformatted using Dolphin software (Dolphin Imaging & Management Solutions, Patterson Technology, USA). Coronal and sagittal images showed severe resorption of alveolar ridge and genial spines enlargement (Figure 3).

**Figure 4:**
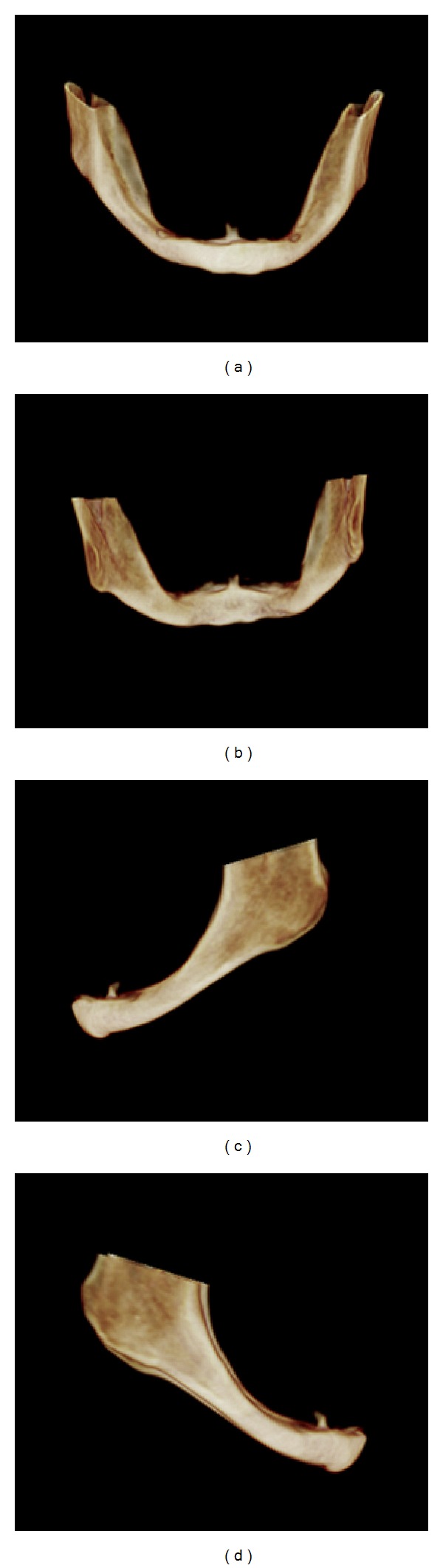
3D reconstructions in which the severe resorption of alveolar ridge and enlargement of the genial spine are clearly seen on frontal (a), posterior (b), left side (c), and right side (d) views.

The genial spine measured about 5.5 mm long by 12 mm wide at its insertion in the bone plate. The 3D reconstructions allowed the volumetric visualization of the anatomic condition (Figure 4).

Therefore, these tomographic images confirmed the diagnosis of severe resorption of the alveolar ridge and enlargement of the genial spines. The chosen treatment was the removal of the lower prosthesis and the reference of the patient to a prosthetic dentistry service.

## 3. Discussion

Genial spines are normally quite small (mean height of 5.82 mm and width of 6.98 mm) [5]; however, if the size is increased, they may lead to a chronic irritation by the poorly adapted prosthesis leading to an anatomic modification and, consequently, interfering with success of mandibular prosthesis [3].

As the patient had no previous radiographic documentation, it was impossible to verify whether she has had the condition all along her life or whether this feature was developed due to the chronic trauma performed by the poorly adapted prosthesis.

During the analysis of the panoramic radiograph, the alteration of the genial spines was not observed due to the superimposition of structures. Occlusal radiograph showed an enlargement of the genial spines. The CBCT images were used to observe both mandibular atrophy and genial spines enlargement, and 3D reconstructions allowed the volumetric visualization of the anatomic condition. Thus, we were able to quantify the extent of the genial spines in this case, which presented width larger than length, sufficient to cause local chronic irritation associated with poorly adapted prosthesis.

Yaedú et al., in 2006 [3], and Gallego et al., in 2007 [4], reported 2 cases of spontaneous fracture of the genial spines. The diagnosis was established using occlusal radiograph and CT images, respectively. The panoramic radiograph could not resolve the diagnosis itself, due to the superimposition of structures. The authors highlighted the importance of occlusal radiograph and CT scan for the evaluation of the genial spines condition. In our reported case, occlusal radiograph and specially CBCT were fundamental for a good visualization of the genial spines, as well as for the determination of the conservative treatment plan. The genial spines were not fractured, which could occur due to normal masticatory forces delivered through mandibular denture [3].

Yin et al., in 2007 [5], used spiral CT to evaluate and measure the genial spines of 40 adult human skulls in comparison with anatomic measurements. The aim of the article was to determine the usefulness of CT for the accurate location of osteotomy in genioglossus advancement procedure. The authors concluded that CT measurements of the genial spines reflect their real anatomy, which is important for the planning of a surgical procedure in the region. In our reported case, we were able to perform an accurate measurement of the genial spines using CBCT images, as well as determining the exact position of the enlarged anatomic structure. The 3D reconstructions in different views were also useful for the volumetric evaluation and spatial visualization of the mandible, which is in accordance with the case reported by Rubira-Bullen et al. [6], in 2010.

## 4. Conclusion

As a conclusion, accurate imaging assessment with the aid of 3D reconstructions allows the elimination of image superimposition and, therefore, plays an important role in the depiction of anatomical and pathological conditions, such as genial spines enlargement.
